# The association of workplace health education with smoking-related behaviour and unequal gains by job position in China: ABWMC programme findings

**DOI:** 10.1186/s13011-021-00392-9

**Published:** 2021-06-30

**Authors:** Haoxiang Lin, Meijun Chen, Yunting Zheng, Qingping Yun, Chun Chang

**Affiliations:** Department of Social Medicine and Health Education, School of Public Health, Peking University Health Science Center, 38 Xueyuan Rd, Haidian District, Beijing, China

**Keywords:** Health education, Smoking-related behaviour, Unequal gains

## Abstract

**Background:**

Although the Chinese government has introduced a series of regulations to promote tobacco-related health education in workplaces, their implementation has been far from satisfactory. The aim of the present study was to explore the association of company-level tobacco-related health education and employee smoking behaviour.

**Methods:**

Data from the 2018 Asia Best Workplace Mainland China programme were used to address these aims. This was a cross-sectional study that included 14,195 employees from 79 companies in mainland China. Spearman correlation tests were used to examine unadjusted correlations between the study variables, and binary logistic regression was used for multivariable analysis. The dependent variables included smoking-related variables or health information-seeking behaviour. The explanatory variable was the company-level tobacco-related health education.

**Results:**

Tobacco-related health education was associated with better smoking harm awareness (OR = 2.23; 95% CI = 1.94–2.56), lower second-hand smoke exposure (OR = 0.73; 95% CI = 0.66–0.81), better perception of the workplace environment (OR = 2.04; 95% CI = 1.84–2.26) and positive health information-seeking behaviour (OR = 2.07; 95% CI = 1.86–2.30). Job position interacted with health education, suggesting that the positive association of health education was lower for general employees than employees who held an administrative position.

**Conclusions:**

Tobacco-related health education is not only associated with lower SHS exposure but also related to more positive environmental perceptions and health attitudes, and these effects are significant for higher-ranking employees. Policy makers should recognize and reduce these potential health disparities.

## Introduction

Smoking is one of the main risk factors driving the growing epidemic of noncommunicable diseases worldwide. Tobacco is on track to claim 200 million lives in China this century, predominantly among the poorest and most vulnerable people [1, 2]. Although the rate of secondhand smoke (SHS) exposure has gradually decreased in China, workplace SHS exposures remain pervasive (i.e., 54.3% in 2015 to 50.9% in 2018) [3, 4]. Company-level tobacco-related health education for employees has contributed to effective tobacco control or SHS exposure reduction strategies [5, 6].

Since the 1980s, the Chinese government has introduced a series of regulations to reduce SHS in workplaces, including smoking bans in public places and support for companies to conduct health education of their employees. However, their implementation has been far from satisfactory [5]. Companies may be reluctant to provide health education due to a lack of time and knowledge. There are only a limited number of companies that have a smoke-free policy and that provide tobacco-related health education, resulting in few studies evaluating workplace health education and its impact on employees’ smoking behaviour.

Prior to implementing potential robust measures to promote smoking-related health education in workplaces, it may be beneficial for policymakers to have a clear understanding of the impact of such activities on Chinese companies. For example, the most common outcomes assessed following health education are knowledge increase and SHS exposure reduction. However, little is known about how such activities are associated with employees’ other behaviours, including their perception of the workplace environment and regular health information-seeking behaviour.

Another relevant problem is unequal gain from tobacco health education. In Western countries, researchers have found that people with different socioeconomic statuses (SESs) but equal health resources have unequal gains [7, 8]. For example, one study reported that African Americans do not gain as much self-rated health as Caucasians with the same resources [8]. Assari and Bazargan found that the protective effects of educational attainment for reducing SHS exposure at work are systemically less for Hispanics than white people [9]. Some scholars believe that the impact of resources on health outcomes is conditional on factors such as SES, poverty and residential segregation [10, 11].

According to the Minorities’ Diminished Returns theory, at least some of the health disparities are due to “less than expected” protective effects, suggesting that population-level health disparities are not all due to resources but also to differential health gains [8, 9]. One recent Chinese study found that families with higher SES have better self-reported health and fewer activities of daily living limitations with the same societal resources [12]. However, there are no previously reported studies on health gain differences for tobacco-related health education. Because of the sustained increase in health status disparities in China over the past half-century, the difference in population-level positive effects from health education has become increasingly important for both scholars and policymakers [13].

The aim of the present study was to explore the association of company-level tobacco-related health education and employee behaviour. Specifically, we formed the following hypotheses: (1) with company-level health education, employees will not only achieve a higher smoking harm awareness and a lower SHS exposure but also show a positive association between their perception of the working environment and health behaviour; and (2) several positive associations of health education will be lower for lower-ranking employees than for higher-ranking employees.

## Methods

### Design and data

Data from the 2018 Asia Best Workplace Mainland China (ABWMC) programme were used to address these aims. ABWMC is an academic/company partnership programme that aims to support companies in building a healthy workplace. The ABWMC programme was designed by Peking University and organized by the American International Assurance Co. All companies may voluntarily join the programme and are free to withdraw at any time. The inclusion criteria were as follows: (1) registered legal companies in China; (2) agreement to participate in the programme; and (3) at least 100 workers who are full-time employees. We used information from baseline employee questionnaires.

The human resource departments of each company delivered the questionnaires to all employees. All employees who (1) were aged 18 years old or above and (2) were full-time employees were invited to participate in this programme. When first opening the link, content related to informed consent was shown, and employees were able to choose whether to complete the questionnaire or quit. If the employees submitted the questionnaire through the link, we assumed that they agreed to participate. The self-check function of the online survey system automatically identified missing data, logical errors and illegal characters.

### Measurements

#### Smoking harm awareness

Smoking harm awareness was measured by the following question: ‘Do you think smoking can cause any of the following diseases? A: stroke, B: heart disease, C: lung cancer, D: cardiovascular disease, E: chronic obstructive pulmonary disease, F: asthma or G: I don’t know.’ Only the participants who chose all answers from A to F were classified as having smoking harm awareness.

#### SHS exposure

In the survey, the participants were asked the following question: ‘How many days a week do you usually suffer from SHS exposure at the workplace for more than 15 minutes? A: almost every day, B: 4-6 days, C:1-3 days or D: never’. Only the participants who chose D were classified as having no SHS exposure.

#### Tobacco-related health education

We defined tobacco-related health education as follows: (1) organized at the company level; (2) all employees had the opportunities to participate; and (3) the content should be related to tobacco control or smoking cessation. This definition was explained to the respondents when conducting the survey.

Such activities were measured by two questions. The participants were asked the following questions: ‘Does your company provide you with tobacco-related health education? A: Yes or B: No’. Respondents who answered ‘Yes’ were then asked ‘Have you ever participated in such activities? A: Yes or B: No’.

We further classified all respondents into three categories: have tobacco-related health education and attend such activities (both of the questions answered ‘Yes’ = 2); have tobacco-related health education but not attending such activities (first question answered ‘Yes’, second answered ‘No’ = 1); and without tobacco-related health education (Otherwise = 0).

#### Perceived workplace environment

There were two variables for this characteristic. The first variable was the employees’ belief that they work in a healthy environment, and the second variable was the employees’ belief that company policies protect their health. For the first variable, the participants were asked the following question: ‘Do you think your working environment is healthy? A: I totally agree, B: I Agree, C: Just ok, D: I do not agree, or E: I totally disagree. Only the participants who chose A and B were classified as believing that they work in a healthy environment.

For the second variable, participants were asked the following question: ‘Do you think your company’s policy can protect your health? A: I totally agree, B: I Agree, C: Just ok, D: I do not agree, or E: I totally disagree. Only the participants who chose A and B were classified as believing that their workplace policies protect the health of employees.

All of the participants needed to answer these questions.

#### Health information-seeking behaviour

In the survey, the participants were asked the following question: ‘How often do you search for health knowledge? A: Always, B: Very often, C: Sometimes, D: Occasionally, or E: Never.’ Only the participants who chose A and B were classified as regularly engaging in this behaviour.

#### Other covariates

We controlled for several variables of individual characteristics, such as gender, age, marital status, education, ethnicity and job position. For job position, we further classified all employees into two categories as follows: administrative employees (participants with administrative rank) and non-administrative employees (participants without administrative rank).

### Data analytical plan

Our data have a hierarchical structure; therefore, we first try to use hierarchical linear modeling by setting individual-level and company-level factors. This type of analysis will take into account the fact that workers’ responses are correlated within companies. Hierarchical linear modeling require that the dependent variable be a continuous variable. Among all of the outcome variables, only SHS exposure can be transferred as a continuous variable. Therefore, we used SHS exposure to conduct such analyses. We ran four standardized models (null model, random coefficients regression model, intercepts as a model, slopes as an outcomes model). However, when we finished the null model, we found that the intraclass correlation coefficient (ICC) was too low (lower than 0.059) and was 0.051, indicating that only approximately 5.1% of the total variation in SHS exposure was attributable to differences among companies/clusters [14]. In other words, we can use the usual method to perform analyses. Therefore, we used logistic regression for our statistics.

Our data analysis was conducted in three steps. First, we examined the distribution of the categorical and continuous variables. Second, Spearman correlation tests were used to examine unadjusted correlations between study variables. Third, we performed binary logistic regression for multivariable analysis.

The dependent variables included smoking-related variables (smoking harm awareness and SHS exposure), working environment variables (perceived workplace environment) and health information-seeking behaviour. The explanatory variable indicated whether the company provided tobacco-related health education (yes = 1 or otherwise = 0).

To examine whether there was an interaction effect between job position and health education, we further conducted regression analysis using two models. Model 1 only entered the main effects of health education, job position and covariates. Model 2 also added an interaction term between job position and health education.

We used SPSS 24.0 (SPSS Inc., Beijing, China) statistical software to conduct all analyses.

### Ethics

All participants were informed that the research team would analyse the data anonymously. This study was approved by Peking University (ethical approval number: IRB00001052–18055).

## Results

### Characteristics of our sample

The total number of participants was 14,195 employees from 79 companies in mainland China. The companies included 51.9% private companies, 32.9% foreign companies, 7.6% state-owned companies, 6.3% joint ventures, and 1.3% other companies. All the companies are indoor workers, and female workers account for 54.9% of the total.

The respondents included 5802 (40.9%) employees who reported working in companies that have tobacco-related health education and 8393 (59.1%) employees who reported working in companies without such activities. Among all of the respondents who reported that their companies had health education, 2317 (39.9%) reported that they did not participate in such activities. Table 1 shows the descriptive statistics of the overall sample.

**Table 1:** Descriptive statistics in the overall sample

Demographics	n/%
**Age**	
16–29	6228(43.9)
30–39	5932(41.8)
40–49	1794 (12.6)
50 and above	241 (1.7)
Mean age (SD)	31.60 ± 7.27
**Gender**	
Male	6408 (45.1)
Female	7787 (54.9)
**Ethnicity**	
Han	13,594 (95.8)
Others	601 (4.2)
**Marriage**	
Single	5534 (39.0)
Married	8491 (59.8)
Divorced or widowed	170 (1.2)
**Education attainment**	
High school/lower	2556 (18.0)
College / above	11,639 (82.0)
**Secondhand smoke exposure**	
Never	6429 (45.3)
1 to 3 days per week	4423 (31.2)
4 to 6 days per week	981 (6.9)
Every day	2362 (16.6)
**Smoking**	
Yes	2955 (20.8)
No	11,240 (79.2)
**Awareness on smoking harm**	
Yes	1779 (12.5)
No	12,416 (87.5)
**Job position**	
Not administrative	9458 (66.6)
Administrative	4737 (33.4)
**Total**	14,195

**Fig. 1:**
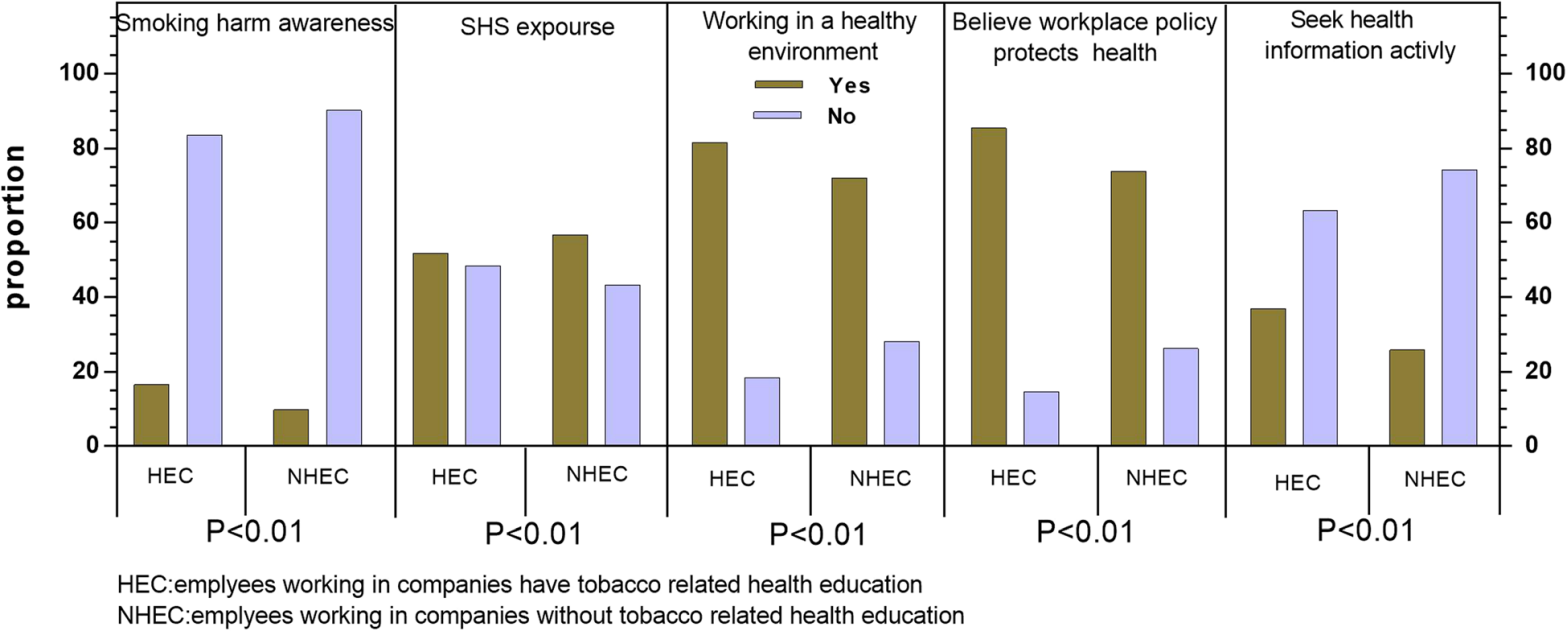
Proportion of employees by health related characteristics

Figure 1 shows the relationship between tobacco-related health education and other key characteristics. Employees in companies with health education reported a lower proportion of SHS exposure, a higher proportion of smoking harm awareness, a higher proportion of a perceived safe workplace environment and more positive health information-seeking behaviour.

### Correlations among the study variables

Table 2 shows the bivariate correlations among the study variables. Health education was positively correlated with smoking harm awareness (Coef. = 0.09; *P* < 0.01), perceived safe workplace environment (Coef. = 0.11; *P* < 0.01) and health information-seeking behaviour (Coef. = 0.12; *P* < 0.01). Moreover, health education was inversely correlated with SHS exposure (Coef. = − 0.05; *P* < 0.01). Education attainment was also associated with positive effects, except SHS exposure.

**Table 2:** Bivariate correlation in the overall sample

Characteristics	1	2	3	4	5	6	7	8
**Heath education**	1.000	0.019*	0.020*	0.099**	−0.050**	0.112**	0.139**	0.118**
**Job position**		1.000	0.121**	−0.002	0.009	0.052**	0.050**	0.043**
**Education attainment**			1.000	0.072**	−0.093	0.115**	0.060**	0.018*
**Smoking harm knowledge**				1.000	0.001	0.031**	0.009	0.106**
**Second-hand smoke exposure**					1.000	−0.106**	−0.085**	− 0.050**
**Believe Working in a healthy environment**						1.000	0.443**	0.078**
**Believe workplace policy protects health of employees**							1.000	0.082**
**Search health information**								1.000

Note: *p < 0.05, **p < 0.01, Spearman correlation test

### The association between health education and smoking harm awareness and SHS exposure

Table 3 presents the summary of the results for both logistic regression models with health education as the independent variable and SHS exposure and smoking harm awareness as the dependent variables. Based on Model 1, health education was associated with better smoking harm awareness (OR = 2.23; 95% CI = 1.94–2.56) and lower odds of workplace SHS exposure (OR = 0.73; 95% CI = 0.66–0.81). No significant interactions were identified between job position and health education for those variables.

**Table 3:** Summary of logistic regression models on workplace SHS exposure and smoking harm awareness

	SHS exposure				Smoking harm awareness			
	Model l (Main Effect)		Model 2 (Interactions)		Model l (Main Effect)		Model 2 (Interactions)	
	OR	95% CI	OR	95% CI	OR	95% CI	OR	95% CI
**Health education**								
Without		Ref		Ref		Ref		Ref
Have but not attend	0.90*	0.82–0.99	0.92	0.82–1.03	1.40*	1.22–1.61	1.47*	1.24–1.74
Have and attend	0.73*	0.67–0.79	0.73*	0.66–0.81	2.11*	1.88–2.36	2.23*	1.94–2.56
**Gender**								
Male		Ref		Ref		Ref		Ref
Female	0.53*	0.50–0.57	0.53*	0.50–0.57	0.96	0.87–1.07	0.96	0.87–1.07
**Age**								
50 and above	0.85	0.64–1.13	0.85	0.64–1.13	1.10	0.70–1.71	1.10	0.70–1.71
40–49	0.77*	0.68–0.88	0.77*	0.68–0.88	1.32*	1.08–1.59	1.32*	1.09–1.60
30–39	0.83*	0.75–0.91	0.83*	0.75–0.91	1.00	0.87–1.16	1.00	0.87–1.16
16–29		Ref		Ref		Ref		Ref
**Ethnicity**								
Han		Ref		Ref		Ref		Ref
Others	0.90	0.76–1.06	0.90	0.76–1.06	0.81	0.62–1.06	0.81	0.62–1.06
**Education**								
High school/lower		Ref		Ref		Ref		Ref
College / above	0.59*	0.53–0.64	0.59*	0.53–0.64	2.00*	1.70–2.36	2.00*	1.70–2.36
**Marriage**								
Single		Ref		Ref		Ref		Ref
Married	1.02	0.93–1.12	1.02	0.93–1.12	0.87	0.76–1.00	0.87	0.76–1.00
Divorced	1.33	0.96–1.85	1.330.96–1.85	0.96–1.85	0.73	0.43–1.25	0.73	0.43–1.25
**Job position**								
Not administrative		Ref		Ref		Ref		Ref
Administrative	1.09*	1.00–1.18	1.11*	1.00–1.22	0.92	0.82–1.03	1.00	0.85–1.17
**Health training×Job position**								
Without				Ref				Ref
Have but not attend			0.93	0.76–1.14			0.86	0.63–1.16
Have and attend			0.97	0.83–1.17			0.85	0.67–1.08

Note: *p < 0.05

Model 1 only entered the main effects of health education, job position and covariates

Model 2 was also added interaction terms between health education and job position

### The association between health education and perceived workplace environment and health information-seeking behaviour

Table 4 presents the summary of the results of both logistic regression models with health education as the independent variable and perceived workplace environment and health information-seeking behaviour as the dependent variables. Based on Model 1, heath education was associated with higher odds of a perceived healthy workplace environment (OR = 2.04; 95% CI = 1.84–2.26) and health information-seeking behaviours (OR = 2.07; 95% CI = 1.86–2.30). Model 2 showed significant interactions between job position and health education with such effects, suggesting that company-level tobacco-related health education has larger positive associations with perceived workplace environment and health information-seeking behaviours for administrative employees than general employees, which was demonstrated by the odds ratio being greater than 1 for the interaction terms.

**Table 4:** Summary of logistic regression models on perceived workplace environment and health information-seeking behavior

	Believe working in a healthy environment				Believe workplace policy protects health				Health information-seeking behavior			
	Model l (Main Effect)		Model 2 (Interactions)		Model l (Main Effect)		Model 2 (Interactions)		Model l (Main Effect)		Model 2 (Interactions)	
	OR	95% CI	OR	95% CI	OR	95% CI	OR	95% CI	OR	95% CI	OR	95% CI
**Health education**												
Without		Ref		Ref		Ref		Ref		Ref		Ref
Have but not attend	1.42*	1.27–1.59	1.28*	1.12–1.45	1.44*	1.29–1.62	1.36*	1.19–1.55	1.15*	1.04–1.28	1.04	0.91–1.18
Have and attend	2.04*	1.84–2.26	1.84*	1.63–2.08	2.87*	2.55–3.23	2.60*	2.27–2.99	2.17*	1.99–2.36	2.07*	1.86–2.30
**Gender**												
Male		Ref		Ref		Ref		Ref		Ref		Ref
Female	1.31*	1.21–1.41	1.31*	1.21–1.42	1.18*	1.09–1.28	1.18*	1.09–1.28	1.29*	1.20–1.39	1.29*	1.20–1.39
**Age**												
50 and above	1.23	0.90–1.67	1.23	0.91–1.67	1.32	0.95–1.85	1.32	0.95–1.85	1.78*	1.34–2.37	1.79*	1.34–2.38
40–49	1.31*	1.13–1.52	1.31*	1.13–1.52	1.30*	1.10–1.52	1.29*	1.10–1.51	1.60*	1.39–1.83	1.59*	1.39–1.83
30–39	1.14*	1.03–1.28	1.14*	1.03–1.27	1.17*	1.05–1.31	1.17*	1.05–1.31	1.15*	1.04–1.27	1.14*	1.03–1.26
16–29		Ref		Ref		Ref		Ref		Ref		Ref
**Ethnicity**												
Han		Ref		Ref		Ref		Ref		Ref		Ref
Others	1.03	0.85–1.26	1.03	0.85–1.25	0.98	0.81–1.20	0.989	0.81–1.21	0.97	0.81–1.16	0.97	0.80–1.16
**Education**												
High school/lower		Ref		Ref		Ref		Ref		Ref		Ref
College / above	1.89*	1.71–2.09	1.86*	1.67–2.05	1.44*	1.29–1.60	1.44*	1.29–1.60	1.19*	1.07–1.31	1.19*	1.07–1.31
**Marriage**												
Single		Ref		Ref		Ref		Ref		Ref		Ref
Married	0.89*	0.80–0.99	0.89*	0.80–0.99	0.96	0.86–1.07	0.96	0.86–1.07	1.00	0.91–1.11	1.00	0.91–1.11
Divorced	0.67*	0.47–0.96	0.67*	0.47–0.96	0.88	0.60–1.28	0.88	0.60–1.28	1.07	0.76–1.50	1.07	0.76–1.50
**Job position**												
Not administrative		Ref		Ref		Ref		Ref		Ref		Ref
Administrative	1.22*	1.11–1.34	1.09	0.97–1.21	1.20*	1.09–1.32	1.10	0.99–1.23	1.12*	1.02–1.20	1.01	0.91–1.13
**Health training×Job position**												
Without				Ref				Ref		Ref		Ref
Have but not attend			1.46*	1.14–1.87			1.24	0.96–1.59			1.36*	1.09–1.68
Have and attend			1.41*	1.12–1.76			1.39*	1.07–1.81			1.15	0.96–1.37

Note: *p < 0.05

Model 1 only entered the main effects of health education, job position and covariates

Model 2 was also added interaction terms between health education and job position

## Discussion

The present study showed that tobacco-related health education is not only associated with lower SHS exposure but also related to more positive environmental perception and health behaviour. Consistent with the predictions, health education was associated with better smoking harm awareness, lower SHS exposure, a better perceived workplace environment and positive health information-seeking behaviour. In addition, the interaction between job position and health education moderated this association.

Changes in knowledge are often targeted because they are recognized as fundamental to changing health behaviour in various behavioural theories [15]. Although this is not an intervention study, we cannot confirm the causal relationship between company-level tobacco-related health education and the various outcomes. However, the information from this study is important and provides evidence from large sample observations. The demonstrated associations are particularly notable given that awareness of tobacco harm is a commonly cited barrier to conducting more active tobacco control measures. If we can address this barrier through health education, the implementation of workplace health promotion measures becomes more practical. However, the positive association with awareness of tobacco harm suggests that educational sessions may be an effective method to increase understanding of a company-level smoke-free workplace policy and encourage compliance with the policy.

Although several studies have found that participation in workplace tobacco-related health education is associated with improved smoking harm awareness and quitting intentions [16], the possible association in the present study was much more comprehensive, resulting in a positive association of health behaviour and health attitude. Therefore, as this study indicated, the implementation of workplace health education may be an opportunity to improve the health of employees at multiple levels.

Although health education has an overall positive association with employee perception of the work environment and health information-seeking behaviour, such associations should not be considered equal between common employees and administrative employees. Some recent studies have found similar patterns for the associations between a wide range of SES indicators and health outcomes [17–19]. In the United States, economic resources and psychological assets systemically result in a smaller health gain for some populations, suggesting that the mechanism generating health disparities is more than differential exposure to resources [8].

It is worth noting, the OR value for the employees who have attended health training is large enough (most of the OR value > 2.0) and larger than the employees who have not attend such training (the OR value are about 1.3) which suggest that the magnitude of the impact is significant at population level. Therefore, we believe that our findings have practical implications. The present study highlights the importance of offering health education at the company level, with the optimal method being to incorporate short-term health education into routine activities. The lack of health education may translate into a ‘missed opportunity’ to promote population health in a cost-effective way. The first step towards the universal adoption of health education provision in workplaces is to educate policymakers and leaders of workplaces. Therefore, the survey report will be disseminated to the participating companies and government agencies, with the aim of encouraging those struggling to catch up and provide accessible options to implement other key health education measures.

Moreover, given the possible existing unequal gain of equal health education, policies that merely focus on the equal distribution of resources and ignore the differential distribution of barriers across groups could be a potential problem. Further studies should explore other health indicators related to this topic because the ultimate objective of such activities is to simultaneously promote health and reduce health disparities. Thus, a related health programme should avoid the unintended effect of exacerbating the existing health inequities rather than reducing them.

It is worth noting that we found that the outcome variables in most models were significantly higher or lower, not only among those participants who reported attending the health education classes but also among those who only reported that this education was adopted in the company. Therefore, it is possible that health education is not the main factor causing the observed effects. For example, if the noncontrolled factor was ‘willingness to show that the company is a good one’, people with higher values of this factor would more likely report that the work environment is healthy and that they are interested in health-related information. In addition, we found that the within-employer variance was almost the same as the total variation (i.e., the ICC was only 0.051 for SHS exposure), suggesting a minimal employer effect overall (regardless of company-level tobacco-related health educational efforts). This finding indicated that some other factors may be at involved. Future research would benefit from using other methods that could identify these hidden factors.

The present study had several limitations. First, we used only cross-sectional data for estimation. What we have revealed were pure associations, not the direct effects of health education. After the ABWMC programme conducts additional follow-up surveys, it will be possible to perform a longitudinal study to obtain more convincing findings. Second, the current study was limited to interested companies, thereby potentially introducing selection bias, and we did not recruit any participants from other areas, such as government employees. Third, our results were based on self-report information. It was not possible to objectively verify the survey answers, and some respondents may not have provided accurate information. Fourth, as the study was not an experimental survey but a cross-sectional survey, it is possible that some other factors caused the observed effects. Fifth, one disadvantage of large samples is that small and even trivial effects can be statistically significant. However, given the relatively small 95% confidence intervals for our main results, we believe that the overall picture is meaningful.

## Conclusion

Taken together, the results of this empirical analysis found that tobacco-related health education is not only associated with lower SHS exposure but is also related to more positive environmental perceptions and health attitudes, which were significant for higher-ranking employees. Policymakers should recognize and reduce potential health disparities.

## Data Availability

The datasets generated and/or analysed during the current study are not publicly available due to ethical restrictions but are available from the corresponding author on reasonable request.

## Abbreviations

SHS: Second-hand smoke
SES: Socio-Economic status
ABWMC: Asia Best Workplace Mainland China
ICC: Intraclass correlation coefficient
